# Hémorragie digestive par expulsion de colle biologique après obturation endoscopique de varice gastrique: à propos d’un cas

**DOI:** 10.11604/pamj.2022.43.75.30034

**Published:** 2022-10-12

**Authors:** Fatimetou Zahra Cheikhna, Nabila Elgasmi, Mohammed Tahiri, Fatima Zahra Elrhaoussi, Fouad Haddad, Wafaa Hliwa, Ahmed Bellabah, Wafaa Badre

**Affiliations:** 1 Service de Gastro-entérologie, Centre Hospitalier Universitaire Ibn Rochd, Casablanca, Maroc

**Keywords:** Hémorragie digestive haute, varice gastrique, hypertension portale, expulsion de colle biologique, cas clinique, Upper gastrointestinal bleeding, gastric varice, portal hypertension, expulsion of cyanoacrylate glue, case report

## Abstract

L´obturation des varices gastriques à la colle biologique est actuellement le traitement de choix des hémorragies digestives par rupture de varices gastriques. L´hémorragie digestive par expulsion de colle biologique après encollage des varices gastriques est une complication rare. Nous présentons une patiente de 65 ans suivie pour cirrhose, en décompensation sous traitement, ayant bénéficié d´une séance d´encollage biologique de varices gastriques 3 mois avant son admission pour hémorragie digestive haute; la fibroscopie œso-gastroduodénale (FOGD) a objectivé des varices œsophagiennes (VO) grade 2 avec une ulcération au sein d´une varice gastrique siège d´un saignement actif en rapport avec l´expulsion de colle biologique. La patiente a bénéficié d´une transfusion sanguine et mise sous sandostatine avec encollage biologique des GOV2 sans incident. L´hémorragie digestive après expulsion de colle biologique est une complication grave de l´encollage dont peu de cas ont été décrits dans la littérature nécessitant le plus souvent un geste endoscopique d´hémostase.

## Abstract (en)

Embolization of gastric varices with cyanoacrylate glue is the treatment of choice for digestive bleeding due to rupture of gastric varices. Gastrointestinal bleeding due to the expulsion of cyanoacrylate glue after gluing of gastric varices is a rare complication. We here report the case of a 65-year-old female patient on cirrhosis and decompensation treatment, undergoing cyanoacrylate glue of gastric varices 3 months before her admission for upper gastrointestinal bleeding; oesogastroduodenal fibroscopy objectified oesophageal varices grade 2 with ulcerated gastric varices and active bleeding due to the expulsion of cyanoacrylate glue. The patient received blood transfusion and sandostatin as well as cyanoacrylate gluing of GOV2 with no complications. Gastrointestinal bleeding after expulsion of cyanoacrylate glue is a serious complication of gluing. Few cases have been described in the literature that required, most often, endoscopic hemostasis.

## Introduction

L´hémorragie digestive liée à l´hypertension portale par rupture de varices est la complication la plus pourvoyeuse de décès au cours de la cirrhose avec une mortalité avoisinant 20% [1,2]. Environ (4%) des patients cirrhotiques ont des varices gastriques objectivées à la fibroscopie oeso-gastro-duodénale de dépistage. Le développement de varice gastrique semble être plus fréquent en cas d´hypertension portale (HTP) non cirrhotique [3]. L´obturation endoscopique par injection de colle biologique est le traitement recommandé pour l´hémostase en urgence et l´éradication des varices gastriques fundiques, qu´il s´agisse de varices fundiques isolées (IGV1) ou en connexion avec les varices œsophagiennes (GOV2) [4]. La sclérose de ces varices par injection de butyl-cyanoacrylate (Histoacryl®) dilué avec le lipiodol en est le traitement de choix. Les varices gastriques sous-cardiales (GOV1) sont habituellement traitées par ligature endoscopique. Cette colle agit comme un adhésif tissulaire qui polymérise au contact du sang dans une varice gastrique. Plusieurs complications ont été rapportées [5], une hémorragie digestive suite à l´expulsion de colle biologique après encollage des varices gastriques est une entité qui est rare. Nous rapportons le cas d´une patiente de 65 ans qui a consulté dans le service de gastroenterogie du Centre Hospitalier Universitaire de Casablanca pour hémorragie digestive haute.

## Patient et observation

**Information sur la patiente:** il s´agit d´une patiente âgée de 65 ans suivie pour cirrhose d´étiologie non encore déterminée (Child B), en décompensation hémorragique et ascitique sous diurétique et bêtabloquant ayant bénéficié d´une séance d´encollage biologique de varices gastriques (3 mois) avant son hospitalisation. Elle a été admise dans notre service pour hémorragie digestive haute faite d´hématémèse et de mélénas.

**Résultats cliniques:** l´examen à l´admission a retrouvé une patiente consciente avec une tension artérielle à 10 /6 cm Hg, une tachycardie à 110 Batt/min, une pâleur cutanéo-muqueuse généralisé. L´examen abdominal a mis en évidence un syndrome d´épanchement péritonéal, et des mélénas au toucher rectal.

**Figure 1:**
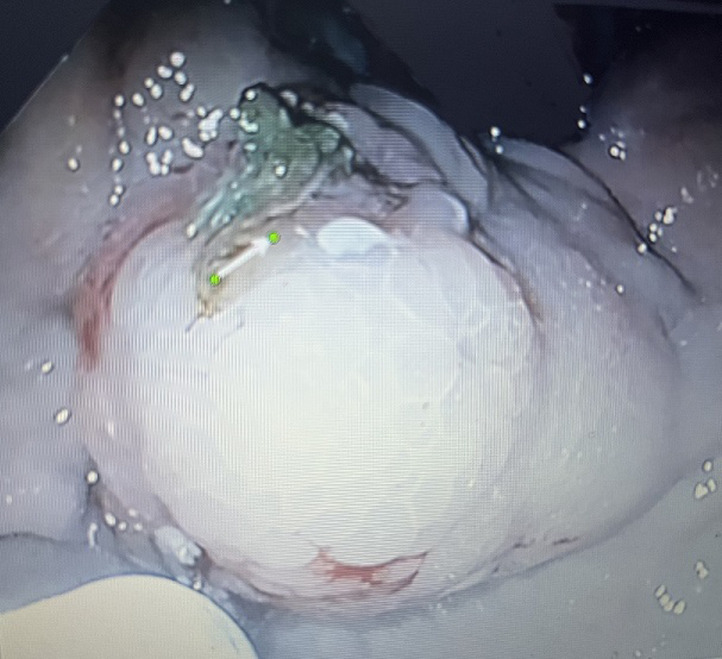
ulcération après expulsion de colle

**Figure 2:**
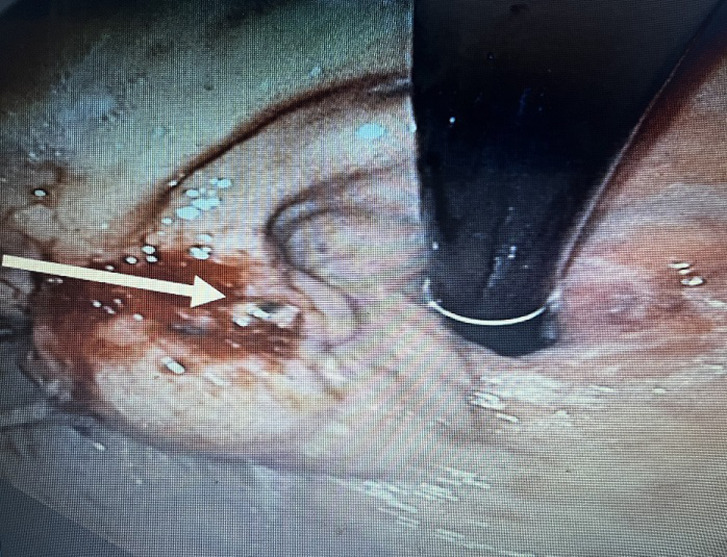
saignement en rapport avec l´expulsion de colle biologique

**Évaluation diagnostique:** le bilan biologique a objectivé une anémie hypochrome microcytaire avec une hémoglobine à 5,5 g /dl, thrombopénie à 57000, TP=58%, facteur V = 44%, bilan infectieux négatif. La FOGD a objectivé des varices œsophagiennes grade 2 sans signe rouge, avec une ulcération au sein d´une varice gastrique (GOV2) (Figure 1), siège d´un saignement actif en rapport avec l´expulsion de colle biologique (Figure 2).

**Figure 3:**
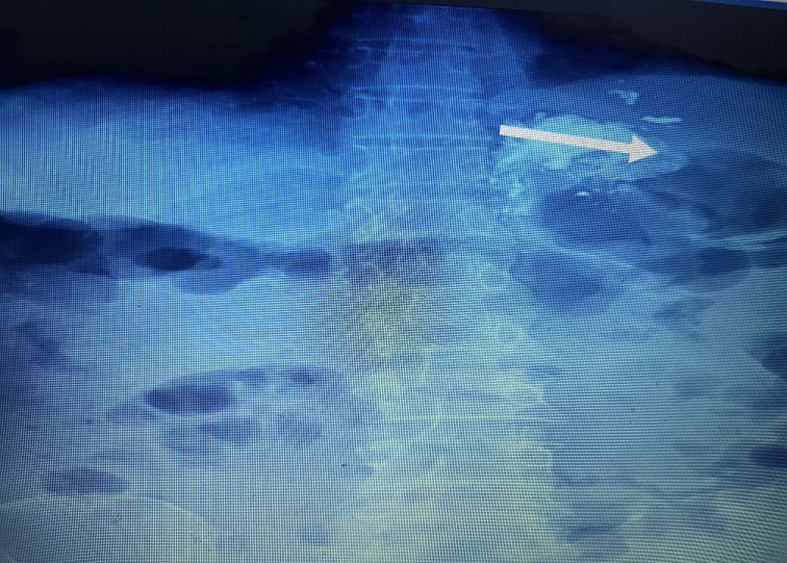
cliché d´abdomen sans préparation réalisé après injection de la colle pour vérifier le bon emplacement du mélange dans le fond de l´estomac

**Intervention thérapeutique:** la patiente a été transfusée de 2 culots globulaires iso groupe et a été mis sous octréotide (sandostatine®) à la dose de 50 µg/h après un bolus de 50 µg. Après stabilisation de son état hémodynamique et traitement vasoactif, elle a bénéficié d´une séance d´encollage biologique par injection d´un mélange contenant 1 ml d´histoacryl et de lipiodol en un seul point de la varice sans incidents immédiat. Un cliché d´abdomen sans préparation a été réalisé pour vérifier le bon emplacement du mélange dans le fond de l´estomac (Figure 3).

**Suivi et résultats:** l´évolution a été marquée par un tarissement du saignement avec une nette amélioration clinique et biologique. Puis la patiente à été déclarée sortante du service au dixième jour de son hospitalisation et a été revue en consultation et ne rapportait aucune plainte.

**Point de vue de la patiente:** pendant son hospitalisation et après la fin du traitement, la patiente était ravie des soins qu´elle a reçus et paraissait optimiste quant à l´évolution de son état.

**Consentement éclairé:** il a été obtenu de la patiente pour que nous puissions utiliser les images. Elle a donné son consentement éclairé pour permettre aux auteurs d´utiliser ses photos pour ce rapport de cas.

## Discussion

L´hémorragie variqueuse par rupture de varices gastrique est une complication rare, mais grave de l´hypertension portale. Elle est estimée par certains auteurs à 10% [6]. L´injection endoscopique du N-butyl-2-cyanoacrylate a été approuvée comme traitement efficace des hémorragies variqueuses gastriques [7]. Ce traitement efficace est tout de même imparfait avec 5 à 10% de récidives et de complications graves, voire fatales [8].

Bureau C *et al*. [9] ont rapporté pour la première fois en 1986 que les saignements dus aux varices gastriques pouvaient être contrôlés par sclérothérapie à l´aide de l´agent adhésif tissulaire (cyanoacrylate de butyle). Depuis lors, plusieurs auteurs ont utilisé différents agents sclérosants pour réaliser l´hémostase des varices gastriques hémorragiques, notamment le cyanoacrylate de N-butyl-2 (histoacryl) [10,11], le cyanoacrylate de 2-octyle [12], l´injection d´oléate d´éthanolamine [13,14], la thrombine [15] et le tétradécylsulfate de sodium [16]. Cependant, le cyanoacrylate de N-butyle 2 est le seul agent prometteur. L´injection de cyanoacrylate peut atteindre une hémostase primaire chez 70% à 95% des patients présentant une hémorragie aiguë de varice gastrique, avec un taux de récidive hémorragique précoce allant de 0% à 28% en 48h [17,18].

Parmi les complications potentielles de l´obturation par colle, la fièvre avec des epigastralgies a été décrite, disparaissant généralement spontanément quelques heures après l´acte. Une translocation bactérienne avec risque de péritonite et de médiastinite a été également rapportée dans la littérature. Une des complications les plus graves, mais très rares, de l´obturation est l´embolie systémique (pulmonaire, cérébrale, splénique...) [1]. Son incidence est estimée à 0,2% [19]. On rapporte également des cas de thrombose extensive (thrombose porte, thrombose de la veine splénique…), Les ulcérations avec expulsion de colle sont tardives, apparaissant 2 semaines à 3 mois après les injections; de ce fait, elles n´exposent pas au risque de récidive hémorragique [20].

L´expulsion de coulée de butyl-2-cyanoacrylate commence généralement 1 mois après le traitement et la majeure partie de l´expulsion de coulée de colle se produit pendant 3 mois après l´injection. Une nouvelle hémorragie après l´injection de colle peut survenir en raison d´une expulsion précoce, ou de la présence d´autres varices non oblitérées [21]. Dans une étude réalisée par Cheng *et al*. [22], axée sur les complications graves de l´obturation endoscopique des varices gastriques avec un adhésif tissulaire, 51 événements indésirables chez 753 patients traités (6,7%) ont été sollicités, et 33 (4,4%) d´entre eux étant des récidives hémorragiques précoces liés à l´expulsion de colle dans les 3 mois. Sharma *et al*. ont rapporté des saignements tardifs d´un ulcère de colle [21]. Choudhuri et al ont identifié une ulcération des varices gastriques chez 32 des 170 patients injectés, mais n´y ont pas attribué de morbidité spécifique [23].

Notre patiente s´est présentée 3 mois après l´encollage des varices gastriques dans un tableau d´hémorragie haute fait d´hématémèse et de mélénas de très grande abondance nécessitant une transfusion sanguine. La FOGD objectivant un saignement actif au sein de la varice encollée en rapport avec l´expulsion de la colle biologique. L´hémorragie abondante après expulsion de colle biologique a été rarement décrite dans la littérature.

## Conclusion

L´obturation des varices gastriques par colle biologique semble être un moyen thérapeutique sûr et efficace, à court et à long terme, en prophylaxie secondaire de l´hémorragie par rupture de varice gastrique. Notre patiente s´est présenté 3 mois après une obturation des varices gastrique dans un tableau d´hémorragie digestive haute secondaire à l´expulsion de la colle biologique, L´hémorragie digestive après expulsion de colle est une complication rare mais grave pouvant mettre en jeu le pronostic vital et il faut y penser devant tout tableau d´hémorragie digestive après encollage biologique de varice et nécessite le plus souvent un geste endoscopique d´hémostase. L´hémorragie abondante après expulsion de colle biologique est une entité rarement décrite dans la littérature.
